# Integrative Trait Analysis for Enhancing Heat Stress Resilience in Tomato (*Solanum lycopersicum* L.): A Focus on Root, Physiological, and Yield Adaptations

**DOI:** 10.3390/plants14040533

**Published:** 2025-02-10

**Authors:** Sharukh Pasha Mohammed, Jo-Yi Yen, Yun-Che Hsu, Hsiu-Yi Chou, Sritharan Natarajan, Assaf Eybishitz

**Affiliations:** 1 World Vegetable Center, Shanhua, Tainan 74151, Taiwan; pasha.sharukh5@gmail.com (S.P.M.);; 2 Department of Crop Physiology, Tamil Nadu Agricultural University, Coimbatore 641003, India

**Keywords:** tomato, heat stress, root traits, morphology, physiology, gas exchange, high-throughput phenotyping

## Abstract

Tomato (*Solanum lycopersicum* L.) is an economically important crop worldwide, particularly in tropical and subtropical regions. However, production is significantly and increasingly affected by the impacts of climate change, including heat and drought stress and emerging pests and diseases. This study specifically evaluated the effects of heat stress on root and shoot morphology, photosynthesis, and yield traits in five tomato genotypes, to identify the characteristics that differentiate heat tolerance from susceptibility. Heat stress experiments were conducted in a polyhouse, one during the summer under high temperatures, with a non-stress trial during the winter under conducive natural conditions. Significant reductions in yield, root traits and photosynthesis were observed across all genotypes under heat stress. However, the genotype MG785-1 maintained a relatively higher yield (298.01 ± 25.1 g), a 37.7% reduction compared to non-stress conditions, while CLN4786F1 showed resilience with a 32.3% decrease compared to its non-stress harvest index. Root dry weight (5.91 ± 0.53 g in MG785-1) and root shoot ratio (0.19 ± 0.01 in MG785-1) were identified as key traits for heat tolerance. Physiological traits, such as photosynthetic rate (11.71 ± 1.61 µmol CO_2_ m^−2^ s^−1^ in MG785-1), were critical for maintaining growth under heat stress. In contrast, the heat-sensitive genotype CLN3961D exhibited a significant decline in yield and physiological performance. Root dry weight and root to shoot ratio were key indicators for heat tolerance, while the photosynthetic rate was critical for maintaining plant growth under stress. These findings underscore the importance of integrated root and physiological traits, providing valuable insights for breeding climate-resilient tomato varieties.

## 1. Introduction

Tomato (*Solanum lycopersicum* L.) is one of the most widely cultivated and consumed vegetable crops globally, valued for its economic value and nutritional content, including vitamins, minerals, and antioxidants such as lycopene [1]. With more than 60% of global tomato production, Asia is the greatest tomato producer, followed by the Americas, Europe, and Africa, contributing 13.4%, 13.5%, and 11.8% of the total tomato harvest, respectively [2]. However, tomato production is increasingly threatened by abiotic stresses, particularly heat stress, which adversely affects growth, yield, and fruit quality. Climate change scenarios indicate that global temperature will rise further, and heatwaves will become more frequent, intense, and prolonged in the future. This may drastically lower tomato yields, and food production and food quality in general [3].

Heat stress is defined as the exposure of plants to temperatures above their optimal range, disrupting various physiological processes [4]. In tomato plants, temperature exceeding 32 °C can impair photosynthesis, reduce pollen viability and hinder fruit set, ultimately leading to significant yield losses [5,6,7]. The optimal mean day/night temperature for tomato is 26/20 °C, depending on developmental stage [8,9]. Temperature only a few degrees above the optimum can reduce fruit production and seed set [10]. The impact of heat stress on tomato plants is multifaceted, affecting morphological and physiological traits of the entire plant. High temperature can lead to reduced leaf area and fruit set, ultimately diminishing overall plant productivity [11]. Heat stress disrupts key processes, such as photosynthesis, reduces chlorophyll content, and accelerates leaf senescence, weakening the plant’s ability to sustain growth and development [12]. Root systems are vital for water and nutrient absorption and are affected by heat stress, reducing root growth and function, and making plants more vulnerable to other stresses [13].

Assessing heat stress tolerance in tomato genotypes has traditionally focused on morphological traits, such as stem diameter, leaf area, days to 50 percent flowering, and fruit set, as well as physiological traits like photosynthetic efficiency and stomatal conductance [14]. While these assessments provide valuable insights, they often overlook traits that are challenging to observe directly, such as root characteristics, which are increasingly recognized as essential determinants of plant resilience under stress conditions due to their role in water and nutrient uptake, hormonal signaling, and overall plant health [15].

The use of advanced phenotyping technologies is accelerating the development of better-adapted varieties to enable agriculture to adapt to shifting climatic conditions and other emerging challenges [16]. Current approaches to analyzing complex plant–environment interactions include proximal and remote sensing plant phenotyping techniques, which utilize multispectral imaging, chlorophyll fluorescence imaging, and gas exchange analysis [17]. These methods, along with continuous, high-throughput monitoring, enable a comprehensive investigation of plant performance and stress responses, offering insights into stress tolerance mechanisms and identifying key traits conferring resilience [18,19]. In this study, it is hypothesized that heat stress significantly affects root architecture, morphological traits, physiological performance, and yield traits in tomato genotypes, with varying degrees of tolerance among genotypes. Specifically, genotypes with robust root systems, efficient photosynthetic rates, and sustained yields, are hypothesized to exhibit superior resilience to heat stress. This study aimed to identify such key traits and genotypes to provide insights into breeding strategies for developing more heat-tolerant tomato lines better suited to future climate challenges.

## 2. Results

For the heat stress trial (Figure S1), temperature data were recorded after transplanting plants into pots, which occurred 30 days after sowing. Throughout the experimental period (mid-June to mid-August), the average day and night temperatures were 34.7 ± 0.26 °C and 27.6 ± 0.17 °C, respectively. These values are significantly higher than the optimal temperature range for tomato cultivation. The consistently elevated temperatures subjected the plants to severe heat stress, potentially impacting their physiological processes and growth. This prolonged exposure highlights the severity of the stress conditions applied during the trial, making it an effective representation of heat stress for assessing plant adaptation and performance.

In contrast, the non-stress trial (Figure S1) captured temperature dynamics from mid-October to mid-December under similar natural conditions in the plastic house. The average day and night temperatures during this period were 26.9 ± 0.31 °C and 21.0 ± 0.47 °C, respectively, which fall within the optimal range for tomato growth and development. These moderate and stable temperatures ensured minimal environmental stress, providing favorable conditions for physiological functions and growth. Thus, the non-stress trial served as a baseline for evaluating plant performance under ideal conditions. Together, these trials demonstrate the contrasting environmental conditions used to assess tomato plant responses to stress and non-stress environments.

Heat stress had a significant impact on morphological, physiological and yield traits in five tomato genotypes, affecting key processes such as the photosynthetic rate (A), stomatal conductance (Gs), and transpiration along with biomass allocation and root traits. ANOVA analysis revealed the sources of variance, highlighting significant interactions between genotype and stress conditions for several traits, including root dry weight (RDW), soil plant analysis development (SPAD), chlorophyll meter values, and harvest index (HI), which underscores the differential responses of genotypes to heat stress (Table 1).

### 2.1. Effect of Heat Stress on Root Morphological Traits of Tomato Genotypes

#### 2.1.1. Root Length (RL)

**Figure 1:**
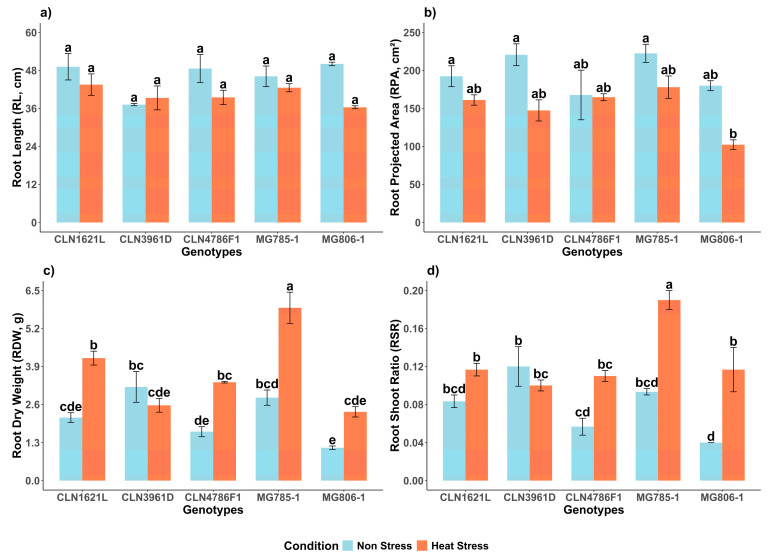
Effects on morphological root traits of tomato genotypes under non-stress and heat stress conditions. (**a**) Root length (RL, cm); (**b**) Root projected area (RPA, cm^2^); (**c**) Root dry weight (RDW, g); (**d**) Root shoot ratio (RSR). Each bar represents the mean ± SE (n = 3); Different letters above the bars represent statistical significance (*p* ≤ 0.05, LSD) and with the same letters indicating no significant difference between the genotypes.

Root length (RL) demonstrated no significant variability among genotypes and between non-stress and heat stress conditions (Figure 1a). Under non-stress conditions, MG806-1 exhibited the longest RL (50.0 ± 0.56 cm), followed by CLN1621L (49.1 ± 4.17 cm), while CLN3961D displayed the shortest RL (37.2 ± 0.37 cm). Notably, under the heat stress condition, CLN1621L maintained the longest RL under stress (43.5 ± 3.39 cm), representing an 11.4% decrease compared to its RL under non-stress conditions. In contrast, MG806-1 showed the lowest RL (36.3 ± 0.46 cm) which corresponds to a 27.4% decrease compared to its RL under the non-stress condition.

#### 2.1.2. Root Projected Area (RPA)

The root projected area (RPA) exhibited significant variability under non-stress and heat stress conditions (Figure 1b). Under non-stress conditions, MG785-1 displayed the highest RPA (222.4 ± 12.0 cm^2^). Heat stress significantly reduced the RPA of MG785-1 to 177.0 ± 14.7 cm^2^, representing a 20% decrease compared to non-stress conditions. In contrast, MG806 exhibited an RPA of 179.92 ± 6.5 cm^2^ under non-stress conditions but showed the most pronounced decline under heat stress, decreasing by 43.2% to 102.18 ± 6.4 cm^2^.

#### 2.1.3. Root Dry Weight (RDW)

Significant differences were observed in root dry weight (RDW) between non-stress and heat stress conditions and among genotypes (Figure 1c). Under non-stress conditions, CLN3961D exhibited the highest RDW (3.20 ± 0.53 g), followed by MG785-1 (2.83 ± 0.26 g). Heat stress caused an overall increase in RDW across most of the genotypes, with MG785-1 recording the highest RDW (5.91 ± 0.53 g), representing a 52.1% increase compared to its RDW under non-stress conditions. In contrast, CLN3961D showed a reduction in RDW under heat stress, declining by 21.5% from 3.20 ± 0.53 g to 2.57 ± 0.24 g.

#### 2.1.4. Root Shoot Ratio

The root shoot ratio (RSR) showed significant variability under non-stress and heat stress conditions (Figure 1d). Under non-stress conditions, CLN3961D displayed the highest RSR (0.12 ± 0.02), followed by MG785-1 (0.09 ± 0.00). Heat stress significantly increased the RSR across all genotypes with MG785-1 exhibiting the highest RSR (0.19 ± 0.01), representing a 52.6% increase compared to its RSR under non-stress conditions. In contrast, CLN3961D showed a decline in RSR under heat stress, decreasing by 16.7% from 0.12 ± 0.02 to 0.10 ± 0.01.

### 2.2. Effect of Heat Stress on Shoot Morphological Traits of Tomato Genotypes

#### 2.2.1. 3D Leaf Area (LA3D)

**Figure 2:**
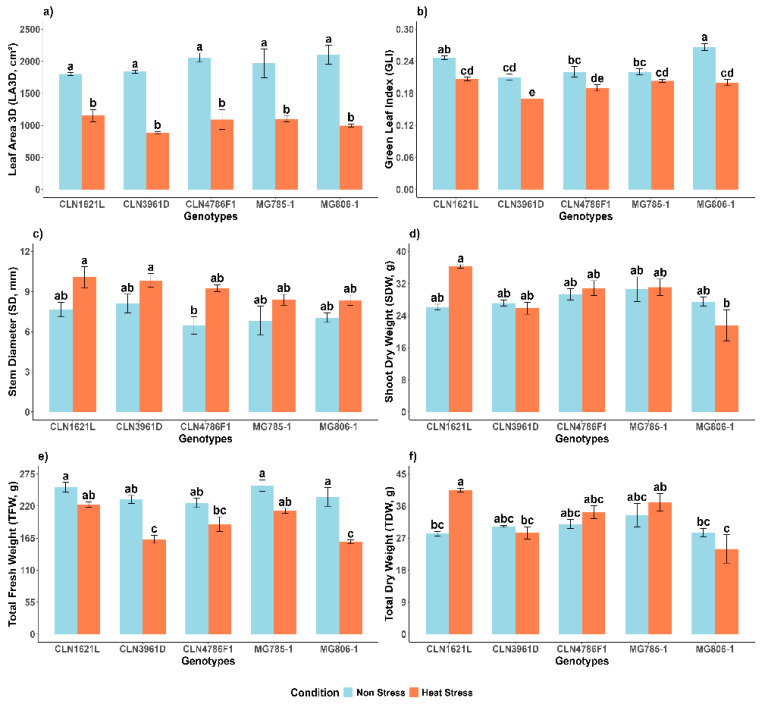
Effects on morphological shoot traits of tomato genotypes under non-stress and heat stress conditions. (**a**) 3D leaf area (LA3D, cm^2^); (**b**) Green leaf index (GLI); (**c**) Stem diameter (SD, mm); (**d**) Shoot dry weight (SDW, g); (**e**) Total fresh weight (TFW, g); (**f**) Total dry weight (TDW, g). Each bar represents the mean value of the trait for a specific genotype and error bars indicate the standard error. Different letters above the bars represent statistical significance (*p* ≤ 0.05, LSD) and with the same letters indicating no significant difference between the genotypes.

The 3D leaf area (LA3D) showed highly significant variation between non-stress and heat stress conditions and no significant variation among genotypes under the same conditions (Figure 2a). Under non-stress conditions, MG806-1 exhibited the largest leaf area (2099.9 ± 148.0 cm^2^), followed by CLN4786F1 (2056.0 ± 70.8 cm^2^), while CLN1621L showed the smallest leaf area (1801.2 ± 19.4 cm^2^). Heat stress led to a substantial reduction in leaf area across all genotypes. Despite this, CLN1621L maintained the highest leaf area under heat stress (1151.8 ± 96.4 cm^2^), representing a 36.0% reduction compared to non-stress conditions. In contrast, CLN3961D was the most affected genotype, reducing from 1838.2 ± 23.2 cm^2^ to 883.42 ± 22.2 cm^2^, corresponding to a 52.0% reduction under heat stress.

#### 2.2.2. Green Leaf Index (GLI)

Green leaf index (GLI) values were significantly impacted by heat stress (Figure 2b). Under non-stress conditions, MG806-1 displayed the highest GLI (0.27 ± 0.01), while CLN3961D showed the lowest (0.21 ± 0.01). Heat stress caused a decline in GLI across all genotypes. Despite the reduction, CLN1621L maintained the highest GLI under heat stress (0.21 ± 0.00), representing a 16.6% decrease compared to non-stress conditions. In contrast, CLN3961D recorded the lowest GLI under stress conditions (0.17 ± 0.00), corresponding to a 19.0% decrease compared to its non-stress values.

#### 2.2.3. Stem Diameter (SD)

Stem diameter (SD) showed resilience to heat stress among the genotypes (Figure 2c). Under non-stress conditions, CLN3961D recorded the largest SD (8.10 ± 0.70 mm), followed by CLN1621L (7.65 ± 0.55 mm). Heat stress caused an increase in SD across most genotypes, with CLN1621L retaining a relatively larger SD (10.08 ± 0.78 mm), representing a 24.0% increase compared to the non-stress condition. In contrast, MG806-1, which recorded the lowest SD under heat stress (8.36 ± 0.38 mm), showed a 15.6% increase compared to its SD under non-stress conditions.

#### 2.2.4. Shoot Dry Weight (SDW)

Under non-stress conditions, MG785-1 exhibited the highest shoot dry weight (30.61 ± 3.07 g), followed by other genotypes with comparable values (Figure 2d). Heat stress did not drastically affect SDW in most genotypes. CLN1621L, in particular, showed resilience with an SDW of (36.3 ± 0.4 g) under heat stress, representing a 28.0% increase compared to the value under non-stress conditions (26.11 ± 0.7 g). In contrast, MG806-1, which exhibited a shoot dry weight of 27.47 ± 1.1 g under non-stress conditions, recorded a 21.3% decrease under heat stress (21.61 ± 3.8 g).

#### 2.2.5. Total Fresh Weight (TFW)

Significant differences were observed in the total fresh weights (TFWs) between the non-stress and heat stress condition and among the genotypes (Figure 2e). TFW was highest in MG785-1 (254.99 ± 10.09 g) and CLN1621L (252.29 ± 8.26 g) under non-stress conditions. Heat stress caused a notable reduction in TFW for all genotypes. Despite the reduction, CLN1621L maintained relatively higher TFW under heat stress (222.82 ± 4.68 g), representing an 11.7% decrease compared to the non-stress condition. In contrast, CLN3961D (163.08 ± 6.9 g) showed a similar reduction of 29.5% under heat stress.

#### 2.2.6. Total Dry Weight (TDW)

Total dry weight (TDW) demonstrated a significant increase under heat stress in most of the genotypes (Figure 2f). Under non-stress conditions, CLN1621L exhibited a TDW of 28.26 ± 0.63 g, which increased to 40.49 ± 0.56 g under heat stress, representing a 30.2% increase. In contrast, MG806-1, which had a TDW of 28.59 ± 1.15 g under the non-stress condition, experienced a drastic reduction, declining to 23.96 ± 3.96 g under heat stress, corresponding to a 16.1% decrease. Among the genotypes, MG785-1 recorded the highest TDW under heat stress (33.44 ± 3.28 g), followed by CLN4786F1 (30.94 ± 1.28 g).

### 2.3. Effect of Heat Stress on Physiological Traits of Tomato Genotypes

**Figure 3:**
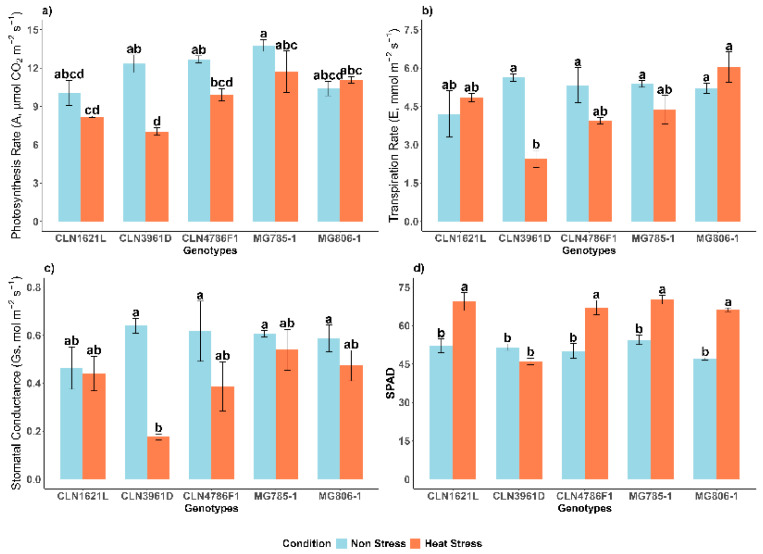
Effects on physiological traits of tomato genotypes under non-stress and heat stress conditions. (**a**) Photosynthetic rate (A, µmol CO_2_ m^−2^ s^−1^); (**b**) Transpiration rate (E, mmol m^−2^ s^−1^); (**c**) Stomatal conductance (Gs, mol m^−2^ s^−1^); (**d**) Soil and plant analysis development (SPAD) under heat stress in tomato genotypes. Each bar represents the mean value of the trait for a specific genotype and error bars indicate the standard error. Different letters above the bars represent statistical significance (*p* ≤ 0.05, LSD) and with the same letters indicating no significant difference between the genotypes.

The bar graphs (Figure 3) represent the variation in physiological traits of different tomato genotypes under non-stress and heat stress conditions. The traits include photosynthetic rate (A), transpiration rate (E), stomatal conductance (Gs), and measurements with a SPAD meter.

#### 2.3.1. Photosynthetic Rate (A)

The photosynthesis rate (A) varied significantly across genotypes and between conditions (Figure 3a). Under non-stress conditions, MG785-1 exhibited the highest photosynthetic rates (A) (13.78 ± 0.46 µmol CO_2_ m^−2^ s^−1^) suggesting superior carbon assimilation, while CLN3961D had significantly lower photosynthetic rates (10.07 ± 0.98 µmol CO_2_ m^−2^ s^−1^). Heat stress caused a decline in photosynthetic rates across all genotypes. Despite the reduction, MG785-1 maintained the highest photosynthesis rate under heat stress (11.71 ± 1.61 µmol CO_2_ m^−2^ s^−1^), representing a 15.0% decrease compared to non-stress conditions. In contrast, CLN3961D exhibited the lowest photosynthesis rate under heat stress (7.04 ± 0.31 µmol CO_2_ m^−2^ s^−1^), corresponding to a 43.0% decrease compared to its non-stress value.

#### 2.3.2. Transpiration Rate (E)

The Transpiration rate (E) exhibited significant differences among genotypes and between conditions (Figure 3b). Under non-stress conditions, the highest transpiration rate was recorded in CLN3961D (5.63 ± 0.15 mmol m^−2^ s^−1^), while CLN1621L had the lowest (4.21 ± 0.90). Heat stress caused variability in transpiration rates among genotypes. MG806-1 demonstrated the highest transpiration rate under heat stress (6.04 ± 0.60 mmol m^−2^ s^−1^), representing a 13.5 increase compared to non-stress conditions, suggesting better evaporative cooling and water regulation. In contrast, CLN3961D exhibited a significant decline in transpiration rates, decreasing by 56.5% from 5.63 ± 0.15 mmol m^−2^ s^−1^ under non-stress conditions to 2.45 ± 0.33 mmol m^−2^ s^−1^, indicating poor water management under stress.

#### 2.3.3. Stomatal Conductance (Gs)

The stomatal conductance (Gs) exhibited less significant differences among genotypes and between conditions (Figure 3c). Under non-stress conditions, values ranged from 0.46 ± 0.09 mol m^−2^ s^−1^ in CLN1621L to 0.64 ± 0.03 mol m^−2^ s^−1^ in CLN3961D. Heat stress caused a notable decline in stomatal conductance across genotypes, MG785-1 maintaining a relatively higher stomatal conductance under stress conditions (0.54 ± 0.09 mol m^−2^ s^−1^), representing a 10% decrease compared to non-stress conditions. In contrast, CLN3961D exhibited the lowest stomatal conductance under heat stress (0.18 ± 0.01 mol m^−2^ s^−1^), reflecting a 72.1% reduction compared to its non-stress value.

#### 2.3.4. Soil and Plant Analysis Development (SPAD-520)

Chlorophyll content, measured as SPAD, increased under heat stress in most genotypes (Figure 3d). Under non-stress conditions, MG785-1 exhibited the highest SPAD value (54.47 ± 1.89) followed by CLN1621L (52.10 ± 2.66), whereas MG806-1 showed the lowest SPAD value (47.0 ± 0.42). Heat stress caused variability in chlorophyll content among genotypes. MG785-1 retained the highest SPAD value under heat stress (70.17 ± 1.72), representing a 10.7% increase compared to the non-stress condition. In contrast CLN3961D experienced a huge decline, with its SPAD value decreasing by 10.5% from 51.45 ± 0.97 under non-stress conditions to 46.03 ± 1.33 under heat stress.

### 2.4. Effect of Heat Stress on Yield Traits of Tomato Genotypes

#### 2.4.1. Number of Fruits Per Plant (NF)

**Figure 4:**
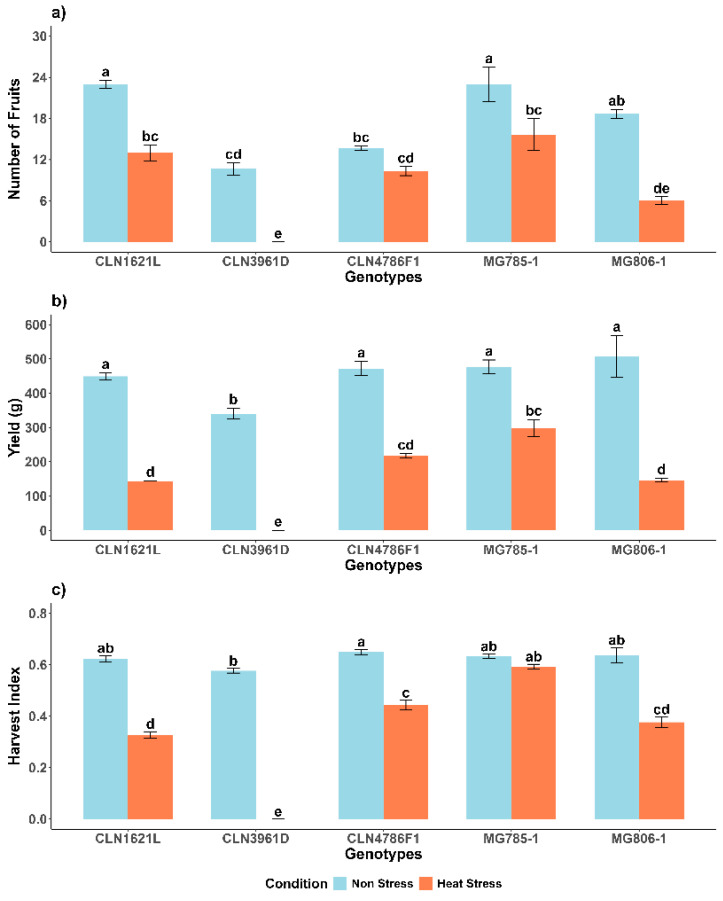
Yield trait measurements of tomato genotypes under non-stress and heat stress. (**a**) Number of fruits per plant (NF); (**b**) Yield (Y, g); (**c**) Harvest index (HI). Each bar represents the mean value of the trait for a specific genotype and error bars indicate the standard error. Different letters above the bars represent statistical significance (*p* ≤ 0.05, LSD) and with the same letters indicating no significant difference between the genotypes.

Under non-stress conditions, genotypes CLN1621L (23 ± 0.58) and MG785-1 (23 ± 2.52) exhibited the highest number of fruits, followed by MG806-1 (18 ± 0.67) and CLN4786F1 (13 ± 0.33), while CLN3961D (10 ± 0.88) produced the lowest fruit count. Heat stress caused a significant reduction in the number of fruits across all genotypes. MG785-1, which maintained a relatively higher fruit count under heat stress (15 ± 2.33), experienced a 32.1% decrease compared to its non-stress condition. In contrast, CLN3961D was the most adversely affected, showing a drastic decline in fruit production under heat stress, reducing from 10 ± 0.88 to no fruits (Figure 4a).

#### 2.4.2. Yield Per Plant (Y)

The yield (Y) under non-stress conditions was highest in MG806-1 (507.24 ± 61.2 g), followed by MG785-1 and other genotypes, while CLN3961D (340.47 ± 15.2 g) recorded the lowest yield. Heat stress significantly reduced yields across all genotypes. Despite the reduction, MG785-1 maintained relatively higher yields under stress (298.01 ± 25.1 g), representing a 37.7% decrease compared to its yield under non-stress conditions, indicating better heat tolerance. In contrast, CLN3961D exhibited negligible yield under heat stress, highlighting its sensitivity to high temperatures (Figure 4b).

#### 2.4.3. Harvest Index (HI)

Under non-stress conditions, the harvest index (HI) was higher than under stress in all genotypes, except CLN3961D, which did not set any fruit. CLN4786F1 (0.65 ± 0.01) and MG806-1 (0.64 ± 0.03) recorded the highest HI values under non-stress conditions. Heat stress caused a notable decline in HI across all genotypes. Despite the reduction, MG785-1 maintained a relatively higher HI under stress (0.59 ± 0.01), representing a 6.3% decrease, Similarly CLN4786F1 showed notable resilience under heat stress, with an HI of (0.44 ± 0.01), representing a 32.3% decrease compared to its non-stress HI. Conversely, CLN3961D exhibited poor performance, with no fruit production under stress (Figure 4c).

### 2.5. Correlation and PCA Analyses

**Figure 5:**
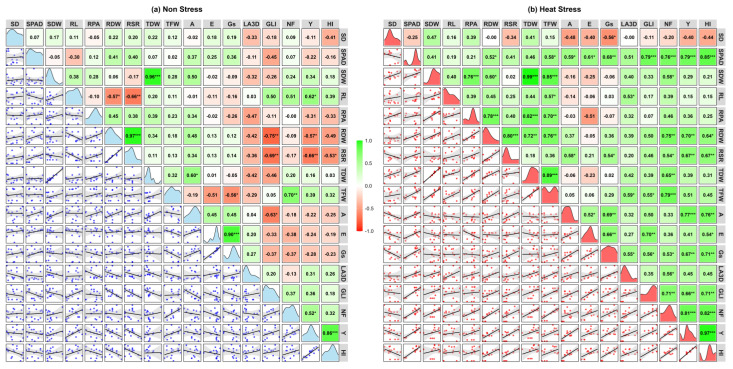
Scatterplot and correlation matrix of tomato traits under (**a**) non-stress and (**b**) heat stress conditions. Green and red boxes show positive and negative correlations, with color intensity reflecting correlation strength. Scatterplots in the lower triangle highlight trait relationships, while density plots on the diagonal show data distribution. *, **, and *** represent significance at 5%, 1%, and 0.1%, respectively. Stem diameter (SD); Soil plant analysis development (SPAD); Shoot fresh weight (SFW); Shoot dry weight (SDW); Root length (RL); Root projected area (RPA); Root fresh weight (RFW); Root dry weight (RDW); Root shoot ratio (RSR); Total fresh weight (TFW); Total dry weight (TDW); Photosynthetic rate (A); Transpiration rate (E); Stomatal conductance (Gs); 3D Leaf area (LA3D); Green leaf index (GLI); Number of fruits (NF); Yield (Y); Harvest index (HI).

Correlation matrices among SD, SPAD, SDW, RL, RPA, RDW, RSR, TDW, TFW, A, E, Gs, LA3D, GLI, NF, Y, and HI for each of non-stress and heat stress environments are shown in Figure 5a,b.

Under non-stress conditions (Figure 5a), most traits showed weak or insignificant correlations, with a few traits with significant negative correlations, indicating minimal variability and interaction and for some traits, such as GLI with RDW and RSR trade-offs. In contrast, heat stress (Figure 5b) induced a notable shift toward positive correlations across physiological, growth, and yield-related parameters. Yield (Y) exhibited strong positive correlations with RDW, RSR, NF, SPAD, A, Gs, and GLI, emphasizing their role in maintaining productivity under stress. SPAD, a measure of leaf greenness, correlated positively with photosynthetic traits (A, Gs, and E) and biomass-related traits, highlighting its importance in sustaining leaf health and productivity under heat stress. Traits like RPA and SDW showed enhanced correlations with TDW and TFW, reflecting their contribution to stress adaptation.

NF shifted to stronger positive correlations with SPAD, RDW, TFW, and GLI, underscoring its critical role in driving productivity under stress. Scatterplots in the lower triangle visually reinforced these stronger correlations, while density plots along the diagonal illustrated greater variability in key traits like SD, A, Gs, E, TFW, and Y under stress. These findings reveal the activation of adaptive physiological and growth mechanisms, enabling tomato plants to counter heat stress and maintain productivity, providing valuable insights into trait interdependence and resilience mechanisms.

**Figure 6:**
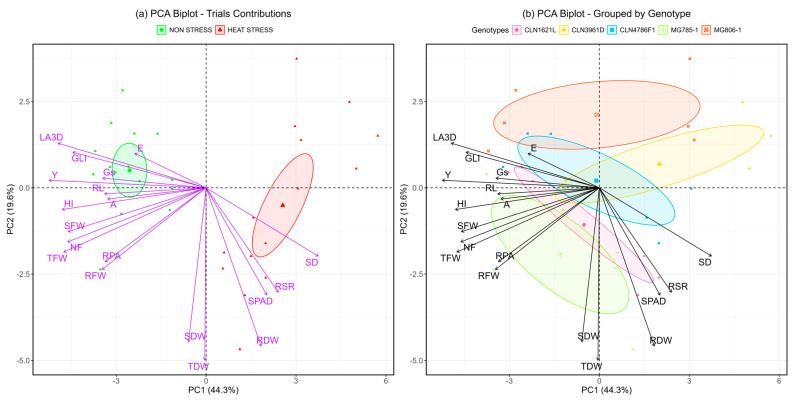
Principal component analysis (PCA) bi-plots showing the contributions of traits and the clustering of treatments and genotypes under non-stress and heat stress conditions. (**a**) PCA bi-plot illustrating the separation of treatments under non-stress and heat stress conditions based on principal components. (**b**) PCA bi-plot grouping genotypes according to their responses to non-stress and heat stress conditions. Stem diameter (SD); Soil plant analysis development (SPAD); Shoot fresh weight (SFW); Shoot dry weight (SDW); Root length (RL); Root projected area (RPA); Root fresh weight (RFW); Root dry weight (RDW); Root shoot ratio (RSR); Total fresh weight (TFW); Total dry weight (TDW); Photosynthetic rate (A); Transpiration rate (E); Stomatal conductance (Gs); 3D Leaf area (LA3D); Green leaf index (GLI); Number of fruits (NF); Yield (Y); Harvest index (HI).

Principal component analysis (PCA), a multivariate statistical tool, was utilized to assess the contributions of various traits under non-stress and heat stress conditions across five tomato genotypes. PCA effectively reduced the dimensionality of the data while preserving most of the variability. The bi-plot (Figure 6) reveals that PC1 and PC2 together account for 64.1% of the total variability, with PC1 contributing 43.4% and PC2 explaining 20.3%.

In the bi-plot for treatments (Figure 6a), PC1 distinctly separated observations into non-stress and heat stress groups. Non-stress samples clustered with higher contributions from traits such as GLI, E, RL, and A, representing physiological efficiency and growth attributes under favorable conditions. Conversely, the heat stress group was closely associated with stress-adaptive traits, including RSR, SPAD, SD, and RDW, highlighting their significant roles in coping with heat-induced challenges.

The genotype-specific bi-plot (Figure 6b) illustrates clear clustering patterns of heat-tolerant genotypes, such as CLN1621L, MG785-1, and CLN4786F1, which were influenced by traits like RSR, SPAD, and SD. These genotypes demonstrated superior adaptive responses to heat stress. On the other hand, the heat-susceptible genotype CLN3961D was positioned closer to the non-stress cluster, reflecting its limited ability to respond to stress conditions. Tolerant genotypes exhibited more resilient physiological and resource allocation mechanisms, enabling better performance under heat stress conditions. These findings provide insights into trait–genotype associations that can inform breeding strategies for heat tolerance in tomatoes.

## 3. Discussion

Breeding for heat tolerance in tomato requires a clear knowledge about how plants respond to high temperature, and which factors limit growth and yield. In particular, information that can contribute to this understanding includes how shoot and root morphology, and physiological factors such as transpiration and photosynthesis, influence yield parameters, including fruit set and size. This study evaluated the effects of heat stress on morphological root and shoot traits, transpiration, photosynthesis, stomatal conductance at the fruit setting stage, and yield traits during harvesting. Consistent heat stress with prolonged exposure to high summer temperature during the day and moderately elevated temperature at night was applied with the aim of assessing heat variation in measured parameters to identify key traits for heat tolerance. To provide a comparison, a non-stress trial was also conducted under conducive winter temperature. The genotypes exhibited varied responses under heat stress and non-stress conditions. Correlation analysis identified key root, physiological, and morphological traits that play a crucial role in adapting to both conditions.

The analysis of root traits under non-stress and high-temperature stress conditions in the polyhouse revealed that certain tomato genotypes exhibited superior performance, particularly in terms of maintaining RDW, RSR, and RPA under heat stress. Genotypes CLN1621L and MG785-1 demonstrated significantly higher RPA and RDW compared to other genotypes. These findings align with previous studies that emphasize the importance of root system architecture in enhancing heat stress resilience [20]. The superior root traits observed in these genotypes could contribute to improved water and nutrient uptake, supporting their resilience under stress conditions [21].

Conversely, CLN3961D exhibited significantly lower values across most root parameters, which likely contributes to its susceptibility to heat stress. These results are consistent with the findings of [9], which suggested that genotypes with less efficient root systems are less capable of withstanding environmental stressors.

In terms of shoot morphological traits, genotypes such as CLN1621L and MG785-1 displayed superior characteristics, including LA3D, GLI, SD, and TDW, which are indicative of better heat tolerance and resilience. SD varies independently and did not contribute to yield in the test panel and other measured parameters. This is supported by research showing that plants with larger SD can better withstand heat stress due to increased structural stability [22,23]. On the other hand, genotypes like CLN3961D, which exhibited smaller leaf area and green leaf index, demonstrated poorer performance under heat stress. The genotypes with higher GLI and greater leaf greenness are better at maintaining photosynthetic efficiency and overall health in stressful environments [24].

Genotypes MG806-1 and MG785-1 exhibited significantly higher photosynthetic and transpiration rates, indicating better carbon assimilation and water regulation under heat stress. Higher photosynthetic rates may contribute to improved resilience, as reported by previous studies [25,26]. In contrast, CLN3961D exhibited significantly lower photosynthetic rates and higher leaf temperatures, reflecting reduced efficiency in photosynthesis and heat dissipation. This finding reinforces the notion that physiological traits such as transpiration rate and stomatal conductance play crucial roles in determining a plant’s ability to withstand heat stress [27].

The genotypes showed significant variation in yield-related traits under heat stress, pinpointing tolerant and susceptible materials. Genotypes MG785-1, CLN4786F1, and MG806-1 exhibited the highest yields, pointing towards their superior tolerance to higher temperatures [28]. The genotypes CLN1621L and MG785-1 exhibited the highest fruit number per plant among the tested genotypes while CLN3961D showed minimal to no yield, underscoring the detrimental effects of heat stress on fruit set in this genotype [12,22]. Tomato breeding is looking for tolerant genotypes that have maintained capacity to allocate resources toward the economically useful part of the plant under stress [29]. The harvest index (HI) varied significantly, with MG806-1 achieving the highest values, indicating efficient resource use under stress [30].

The correlation analysis (Figure 5) illustrates intricate relationships among various growth and yield parameters in tomato genotypes subjected to non-stress and heat stress. Under heat stress, significant positive correlations were observed between photosynthesis rate (A), transpiration rate (E), and stomatal conductance (Gs), suggesting coordinated gas exchange mechanisms that support photosynthesis under stress [31]. Additionally, LA3D was strongly correlated with TDW and GLI, indicating the importance of leaf area and greenness for maintaining biomass and photosynthetic efficiency under heat stress [32]. A significant positive correlation between plant biomass traits and root projected area indicates the critical role of the root system in supporting biomass production, consistent with findings by [33], who emphasized the importance of root architecture in nutrient uptake and stress resilience. Additionally, the strong correlation between yield and the number of fruits underscores the pivotal role that fruit number plays in determining overall yield, reinforcing the concept that reproductive success is closely tied to vegetative health [29]. Under non-stress conditions, correlations were more stable, with moderate positive correlations between LA3D and TDW. However, a negative correlation between photosynthetic rate (A) and GLI suggests a trade-off between photosynthesis and leaf greenness in optimal conditions [34].

The positive correlations between photosynthetic rate (A), stomatal conductance (Gs), and transpiration rate (E) highlight the crucial role of stomatal activity in promoting photosynthesis, especially under heat stress. These findings align with previous research linking stomatal function to plant performance in challenging conditions. Several correlations were found to be non-significant, such as that between stomatal conductance (Gs) and RL; these results indicate the complexity of interactions among traits under non-stress and heat stress, which warrants further investigation to understand the underlying mechanisms. These results demonstrate that heat stress amplifies trait correlations, reflecting adaptive mechanisms for stress tolerance, while non-stress conditions show more stable trait relationships. This highlights the importance of coordinated physiological and morphological traits in stress resilience.

The presented results inform both the agronomic performance of five tomato genotypes under heat stress and the morphological and physiological differences among the genotypes, which may contribute to producing more or less yield under heat stress. The correlations between the trait values help generate a hypothesis regarding the responses that contribute to greater yield in the test panel. This analysis revealed that under non-stress conditions, genotypes exhibited physiological traits associated with efficient growth, including higher GLI, transpiration rate (E), RL, and photosynthesis rate (A). These traits reflect the plant’s ability to maintain healthy growth and efficient metabolic function in optimal environments [35]. Under heat stress conditions, the PCA bi-plot (Figure 6a) clearly segregated the samples, with heat stress conditions clustering with stress-adaptive traits, such as the root shoot ratio, SPAD chlorophyll content, stem diameter, and root dry weight. These traits are critical for managing water and nutrient availability under stress and are typically associated with heat tolerance mechanisms in plants [36]. The clustering pattern indicates that heat-adaptive genotypes, such as CLN1621L and MG785-1, rely heavily on these traits to withstand elevated temperatures, maintaining higher photosynthetic capacity and biomass under heat stress [37].

The genotype-specific PCA bi-plot (Figure 6b) demonstrated that heat-tolerance in CLN1621L, MG785-1, and CLN4786F1 was closely associated with traits like SPAD, SD, and RSR, highlighting their superior capacity for coping with heat stress. These genotypes exhibit enhanced physiological responses, including improved water use and nutrient allocation, which support better performance under high-temperature conditions [36]. In contrast, CLN3961D, a heat-sensitive genotype, clustered closer to the non-stress group, underscoring its limited adaptability to heat stress. This genotype showed poorer physiological responses under heat stress, reflecting its vulnerability to environmental challenges [38]. The clustering of genotypes based on these traits offers valuable insights into their adaptive mechanisms and can guide breeding programs aimed at improving heat tolerance in tomatoes. The results indicate that genuine heat stress conditions were present during the trial, providing strong evidence for this. These findings are consistent with previous studies that confirm the impact of heat stress on traits like root growth, photosynthesis, and transpiration [14].

## 4. Materials and Methods

### 4.1. Plant Material and Experimental Design

The experiment was conducted in a polyhouse at the World Vegetable Center, Taiwan (latitude 24°57′31.392″ N, longitude 121°12′29.772″ E). Seeds of all tomato lines were sown in May 2024 for the heat stress trial and in September 2024 for the non-stress trial. The seeds were sown in 72-well plastic trays filled with seedling peat, a growing substrate manufactured by Stender AG (Schermbeck, Germany). The substrate had a pH range of 5.5–6.5, 90% organic matter, 1.0% total nitrogen, 0.1% total phosphorus, 0.2% total potassium, 67.8% moisture content, and an electrical conductivity (EC) of 0.7 dS/m. Prior to sowing, seeds were disinfected using trisodium phosphate (TSP) and hydrochloric acid (HCl). *Trichoderma* was applied two days after sowing to prevent diseases and promote seedling growth.

Seedlings were raised under controlled conditions in a nursery house at WorldVeg. After 30 days of sowing, the seedlings were transplanted into 18 cm × 18 cm plastic pots in mid-June for the heat stress trial and mid-October for the non-stress trial. The pots were filled with sterilized field soil consisting of sand, silt, and organic matter to ensure uniform growing conditions. To avoid water-limiting conditions, the plants were well-irrigated throughout the growth cycle.

Both experiments were conducted under identical agronomic practices to ensure consistency, including regular irrigation, fertilization, pest control, and management practices. Fertilizer Tai-fat No. 43 (manufactured by Taiwan Fertilizer Co., Ltd., Taipei, Taiwan) was applied every two weeks to ensure optimal nutrient availability. The only variable was temperature, with the heat stress trial conducted from mid-June to mid-August, being the hottest period in Taiwan, to simulate high-temperature stress conditions, and the non-stress trial conducted from mid-October to December, a period with optimum temperatures for tomato growth and development.

The experiment followed a completely randomized design (CRD) with factorial arrangements based on the tomato genotypes. This design allowed for the isolation of temperature effects on the performance of the tomato genotypes.

Five tomato genotypes (Table 2) were used in this study. These genotypes were developed by the World Vegetable Center (WorldVeg) for their heat tolerance. Among them, CLN3961D serves as a susceptible check, while CLN1621L is used as a heat-tolerant check. These genotypes were evaluated to compare root system performance and physiological and morphological responses under different environmental conditions.

The experiment was conducted under two contrasting temperature regimes: a heat stress trial from mid-May to mid-August and a non-stress trial from mid-September to mid-December, both under natural conditions in a plastic house. The temperatures were recorded using data loggers in both the trials.

### 4.2. Root Trait Measurements

#### Root Washing Technique

**Figure 7:**
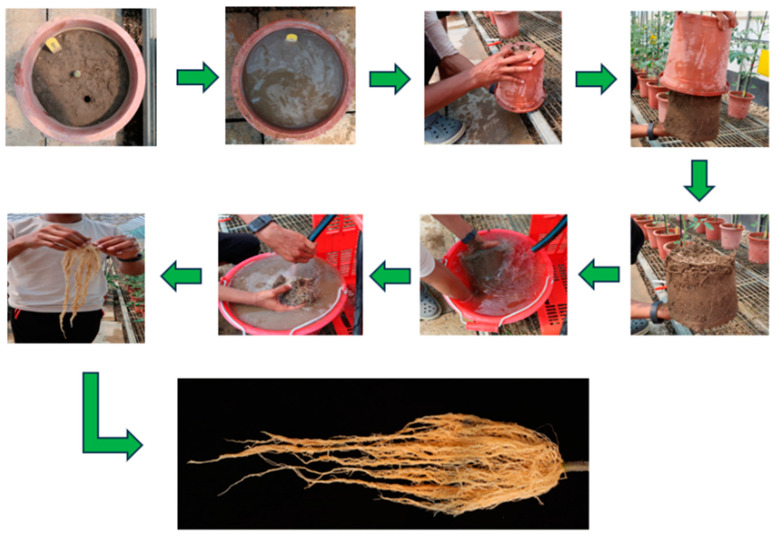
Root washing technique for isolating and cleaning root systems. Plants were excised above the soil, soil–root clumps were saturated and inverted, and roots were washed gently with water to preserve integrity for further analysis.

To assess root morphology and biomass under heat stress and non-stress conditions, a modified root washing technique based on the method described by [39] was employed to prevent damage to the delicate root structures. The above-ground parts of the plants in different growth stages were excised at 1cm from the soil surface to facilitate the isolation of the root system. Pots containing the root–soil samples were thoroughly irrigated and allowed to soak for 10 min. After the soil was fully saturated, the pots were carefully inverted to release the soil–root clumps. The root system samples were then immersed in water and the soil was gently washed away using a light stream of water from a hose, ensuring the integrity of the root structure. The cleaned roots were subsequently used for further analysis (Figure 7). The different root traits measured included RL, RPA, RDW, and RSR. Root length and root projected area were taken at the 50% fruit setting stage.

The washed roots were spread out on a flat surface and digital images were captured using a Canon G7X camera (Canon Inc., Tokyo, Japan). The images were subsequently analyzed using ImageJ© software (version 1.53e) [40] to quantify RL (cm) and RPA (cm^2^). RDW (g) was recorded after oven drying using a precision balance [41]. The RSR was calculated by dividing the dry weight of the roots by the dry weight of the shoots (Section 4.3.3), determining the allocation of biomass between the root and shoot systems.

### 4.3. Phenotyping

#### 4.3.1. Digital Phenotyping

Digital phenotyping was carried out using the Trait Finder system with two Plant Eye 3D scanners (Plant Eye F500, Phenospex, Heerlen, The Netherlands) for collecting multispectral data at 50% fruit set with three replications. The reflection of light in several spectral bands can detect potential variations in spectral properties of plants LA3D and GLI.

#### 4.3.2. Stem Diameter

SD (mm) was measured at the base of the stem, just above the soil surface, using a digital caliper (CD-20APX, Mitutoyo Co., Ltd., Kanagawa, Japan) with a precision of 0.01 mm to ensure high measurement accuracy. Each plant’s stem diameter was measured in three different locations at the base of the stem to account for potential variations in the stem shape and thickness. These triplicate measurements were taken for each plant to minimize measurement errors and provide a more reliable estimate of stem thickness.

#### 4.3.3. Total Dry Weight

The TDW (g) was calculated by adding the shoot dry weight and root dry weight. Both parts were dried in an oven at 80 °C for 48 h until reaching a constant weight before measurement.

### 4.4. Physiological Measurements

#### 4.4.1. Gas Exchange Parameters

The measurements were conducted using a portable photosynthetic system (LI-6800, LICOR Biosciences, Lincoln, NE, USA) between 10:00 a.m. and 2:00 p.m. at the 50% fruit setting stage. Gas exchange parameters namely, photosynthetic rate (A, µmol CO_2_ m^−2^ s^−1^), stomatal conductance (Gs, mol m^−2^ s^−1^), and transpiration rate (E, mmol m^−2^ s^−1^) were measured on the third fully expanded leaf of 5 tomato genotypes with three replications per genotype.

#### 4.4.2. SPAD

The non-destructive chlorophyll content index was measured as an average of three leaves per plant using a SPAD meter (SPAD-502 Plus, Minolta, Osaka, Japan) at 50% fruit set. The average of the three plants measurements was used for analysis.

### 4.5. Yield Traits and Harvest Index

The yield (Y, g), number of fruits per plant (NF), and harvest index (HI) were measured during the harvesting stage. The harvest index was calculated as the ratio of the economic yield (weight of harvest fruits) to the total plant biomass.

### 4.6. Statistical Analysis

Statistical analysis was performed to evaluate the physiological, morphological, root, and yield traits under non-stress and heat stress conditions across different genotypes. Two-way ANOVA, performed using base R [42], was applied to assess the effects of genotype, condition, and their interaction on each trait. Post hoc pairwise comparisons were conducted using the *agricolae* package with the Hochberg-adjusted Least Significant Difference (LSD) test to determine significant differences between treatment combinations, with grouping letters indicating homogeneity. The mean and standard error were computed for all traits using *dplyr* and bar plots with error bars and grouping letters generated using *ggplot2*. Correlation matrix scatterplots and principal component analysis (PCA) bi-plots were constructed using the *ggpairs* and *fviz_pca* function.

## 5. Conclusions

This study highlights the critical traits contributing to heat stress tolerance in tomato, offering valuable insights for breeding programs aimed at developing resilient varieties. Genotypes such as MG785-1, MG806-1, and CLN4786F1 exhibiting superior root system traits, exhibited enhanced photosynthetic efficiency, and sustained yields under heat stress conditions. Root traits like the root dry weight and the root shoot ratio played pivotal roles in water and nutrient uptake, while physiological traits like the photosynthetic rate and SPAD values supported carbon assimilation and overall leaf health. In contrast, the heat-sensitive genotype CLN3961D demonstrated significant yield reductions and poor physiological performance, underscoring the importance of improving stress adaptability. Correlation and principal component analysis analyses revealed strong linkages between root, physiological, and yield traits under heat stress, emphasizing the value of integrated traits for resilience. These findings represent an important step forward in providing a framework for breeding better-adapted and climate-smart tomato varieties that are more capable of sustaining productivity under rising temperatures in future climate scenarios.

## Figures and Tables

**Table 1:** Analysis of variance (ANOVA) results for morphological, physiological, and yield traits under heat stress and non-stress conditions.

Parameter	Source of Variation		
	Genotype (G)	Conditions (C)	Interaction (G × C)
Stem diameter (SD)	NS	***	NS
Soil plant analysis development (SPAD)	***	***	***
Shoot fresh weight (SFW)	**	***	*
Shoot dry weight (SDW)	**	NS	*
Root length (RL)	NS	**	NS
Root projected area (RPA)	*	***	NS
Root fresh weight (RFW)	***	***	NS
Root dry weight (RDW)	***	***	***
Root shoot ratio (RSR)	***	***	**
Total dry weight (TDW)	**	NS	*
Total fresh weight (TFW)	***	***	NS
Photosynthetic rate (A)	**	***	**
Transpiration rate (E)	NS	*	**
Stomatal conductance (Gs)	NS	***	NS
3D leaf area (LA3D)	NS	***	NS
Green leaf index (GLI)	***	***	**
No. of fruits (NF)	***	***	*
Yield (Y)	***	***	**
Harvest index (HI)	***	***	***

* = Significant at 5% level; ** = Significant at 1% level; *** = Significant at 0.1% level; NS = Non-significant.

**Table 2:** Tomato genotypes analyzed in this study.

S. No.	Genotype	Growth Habit	Origin
1	CLN1621L	Determinate	WorldVeg, Taiwan
2	CLN3961D	Semi determinate	WorldVeg, Taiwan
3	CLN4786F1	Semi determinate	WorldVeg, Taiwan
4	MG806-1	Semi determinate	WorldVeg, Taiwan
5	MG785-1	Semi determinate	WorldVeg, Taiwan

## Data Availability

Data are contained within the article.

## Supplementary Materials

The following supporting information can be downloaded at: https://www.mdpi.com/article/10.3390/plants14040533/s1, Figure S1: Temperature profiles during the experimental period for (a) Heat Stress Trial (mid-June to mid-August) and (b) Non-Stress Trial (mid-October to mid-December) under natural conditions in a plastic house. Figure S2: Phenotypic representation of the tomato genotype MG785-1 under heat stress conditions. The images depict the overall plant morphology, root architecture with a robust root system, fruit development on the plant, and harvested fruits. Measurements of root length and fruit size are shown with a ruler for scale. Figure S3: Phenotypic representation of the tomato genotype MG806-1 under heat stress conditions. The images show overall plant morphology, root architecture with a ruler for scale, and harvested fruits with a ruler for scale to demonstrate fruit size and shape. Figure S4: Phenotypic representation of the tomato genotype CLN4786F1 under heat stress conditions. The images depict the plant morphology, detailed root system architecture with a ruler for scale, and harvested fruits showcasing size and shape variations under heat stress conditions. Figure S5: Phenotypic representation of the tomato genotype CLN1621L under heat stress conditions. The images display plant morphology, detailed root system structure with a ruler for scale, and harvested fruits highlighting size and uniformity under stress conditions. Figure S6: Phenotypic representation of the tomato genotype CLN3961D under heat stress conditions. The images showcase plant morphology, detailed root system architecture with a scale, highlighting root length and structure, and the overall plant performance under stress condition.
