# Supplementary material for: Integrative Trait Analysis for Enhancing Heat Stress Resilience in Tomato (Solanum lycopersicum L.): A Focus on Root, Physiological, and Yield Adaptations
Source: Plants (Basel). 2025 Feb 10;14(4):533. doi: 10.3390/plants14040533 (PMC11858947; doi:10.3390/plants14040533)

Figure S1. Temperature profiles during the experimental period for (a) Heat Stress Trial (mid-June to mid-August) and (b) Non-Stress Trial (mid-October to mid-December) under natural conditions in a plastic house.

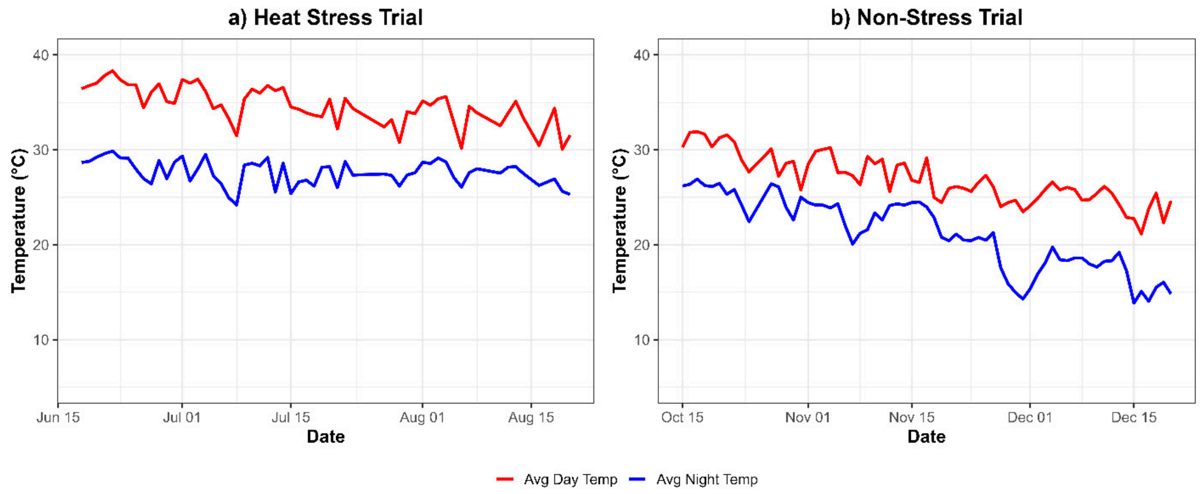

Figure S2. Phenotypic representation of the tomato genotype MG785-1 under heat stress conditions. The images depict the overall plant morphology, root architecture with a robust root system, fruit development on the plant, and harvested fruits. Measurements of root length and fruit size are shown with a ruler for scale.

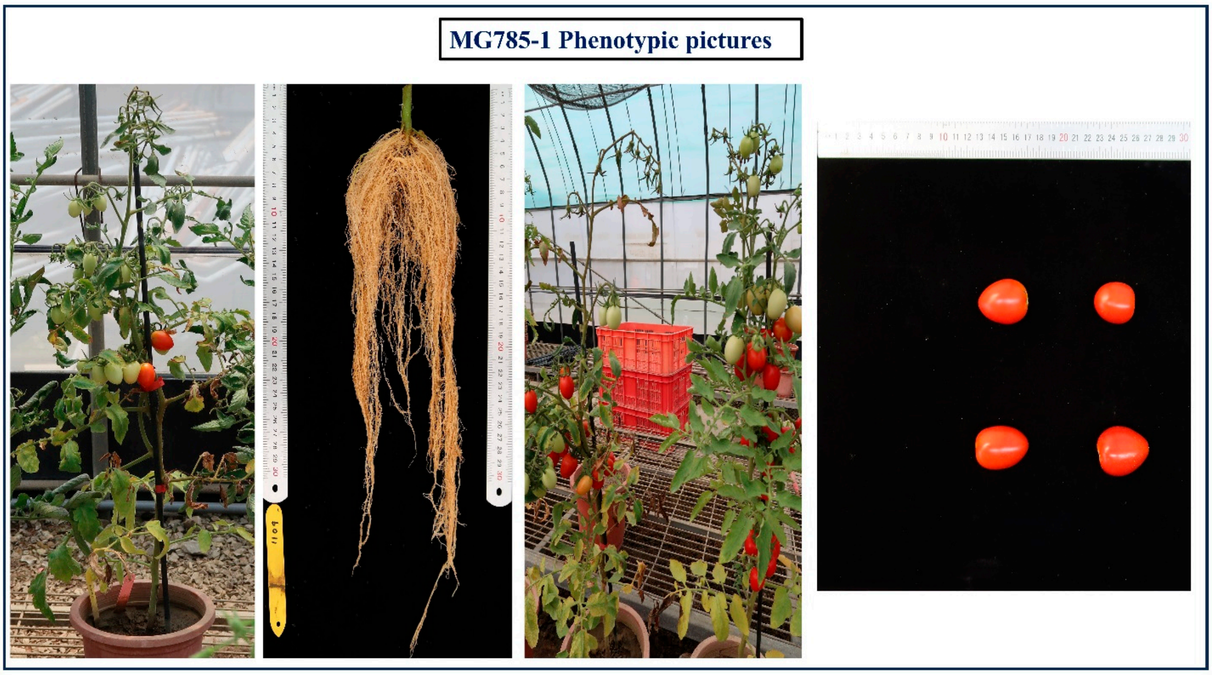

Figure S3. Phenotypic representation of the tomato genotype MG806-1 under heat stress conditions. The images show overall plant morphology, root architecture with a ruler for scale, and harvested fruits with a ruler for scale to demonstrate fruit size and shape.

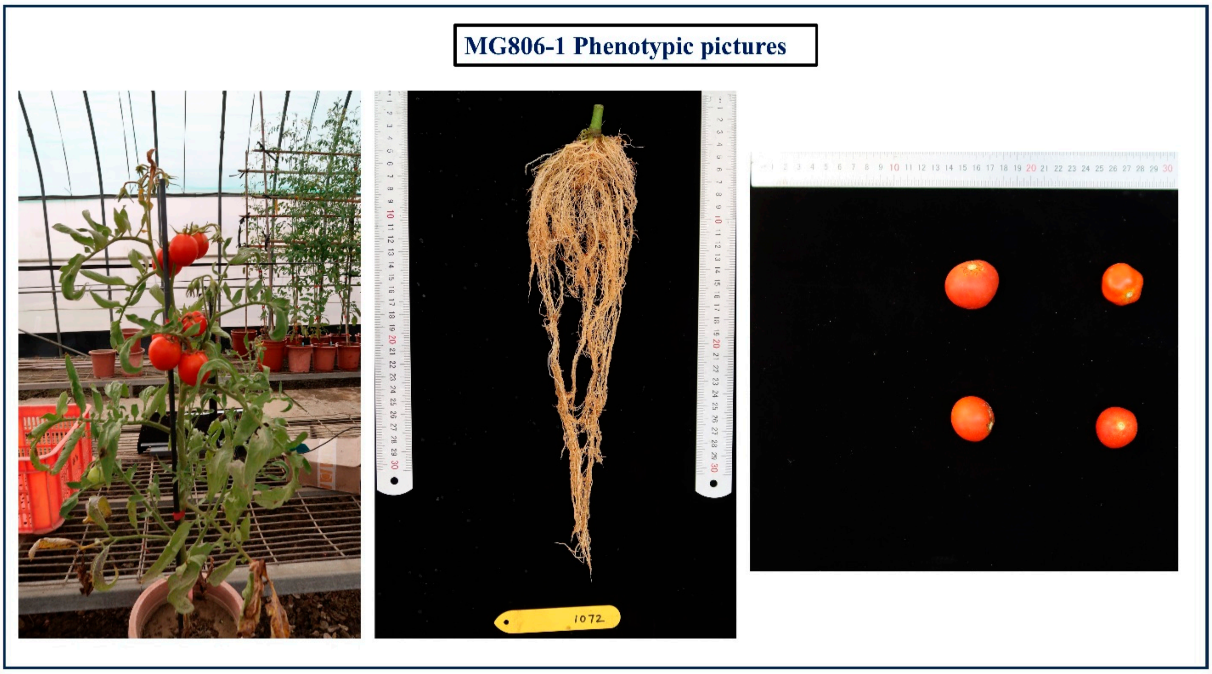

Figure S4. Phenotypic representation of the tomato genotype CLN4786F1 under heat stress conditions. The images depict the plant morphology, detailed root system architecture with a ruler for scale, and harvested fruits showcasing size and shape variations under heat stress conditions.

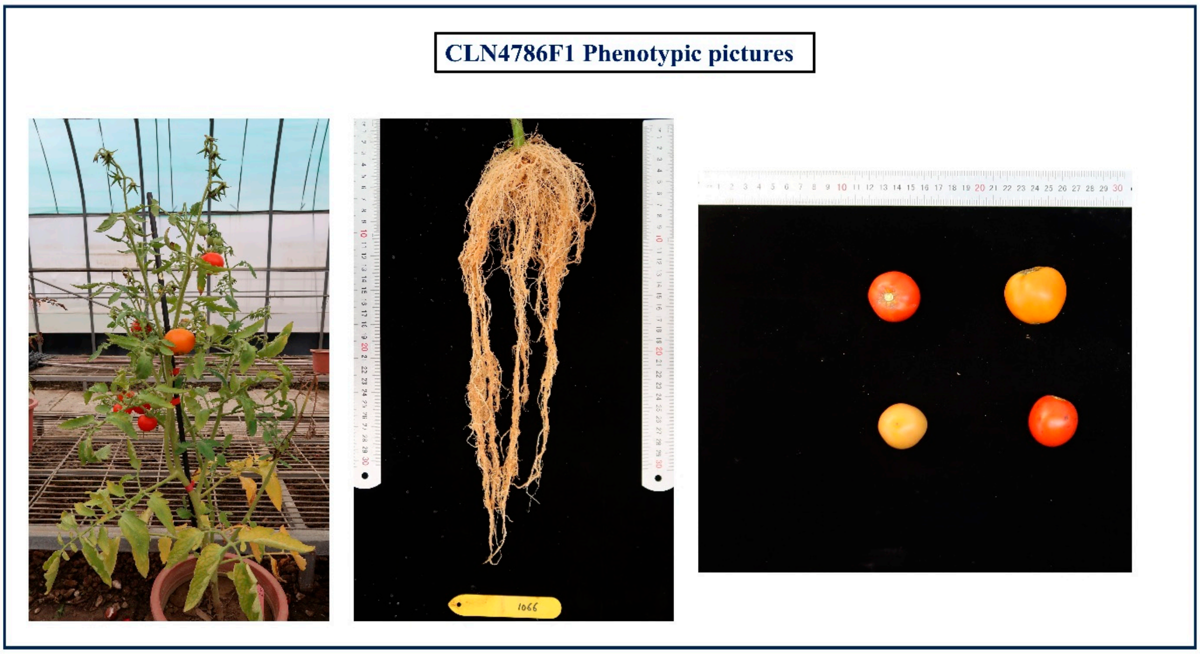

Figure S5. Phenotypic representation of the tomato genotype CLN1621L under heat stress conditions. The images display plant morphology, detailed root system structure with a ruler for scale, and harvested fruits highlighting size and uniformity under stress conditions.

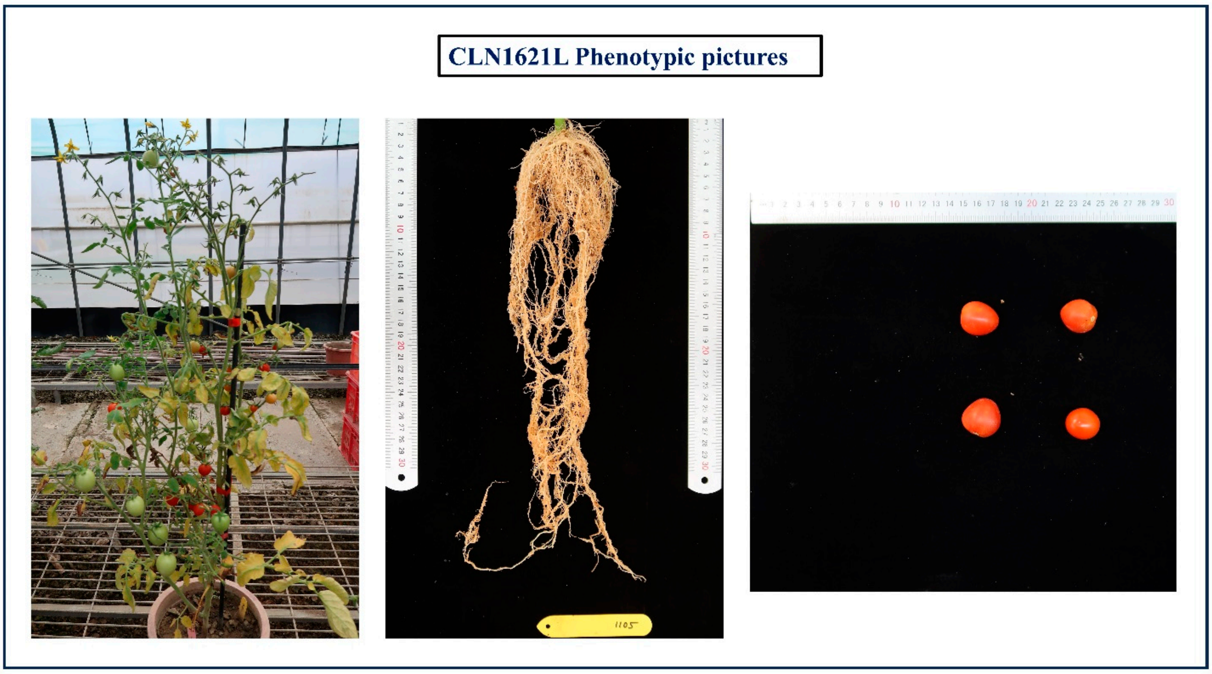

Figure S6. Phenotypic representation of the tomato genotype CLN3961D under heat stress conditions. The images showcase plant morphology, detailed root system architecture with a scale, highlighting root length and structure, and the overall plant performance under stress condition.

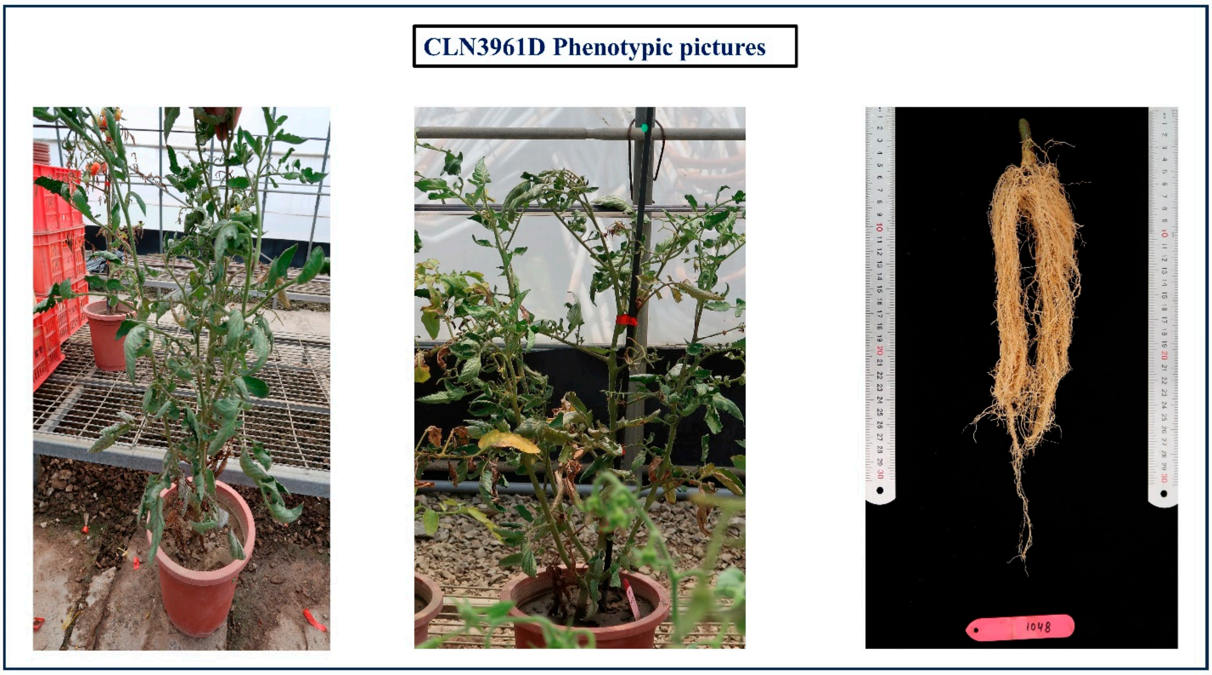

Supplement: Supplementary file 1 [file plants-14-00533-s001.zip › plants-3429652-supplementary.pdf]
